# 177 Lu-PSMA-617 radioligand therapy of metastatic castration-resistant prostate cancer: Initial 254-patient results from a prospective registry (REALITY Study)

**DOI:** 10.1007/s00259-021-05525-7

**Published:** 2021-09-07

**Authors:** Fadi Khreish, Zaidoon Ghazal, Robert J. Marlowe, Florian Rosar, Amir Sabet, Stephan Maus, Johannes Linxweiler, Mark Bartholomä, Samer Ezziddin

**Affiliations:** 1 Department of Nuclear Medicine, Saarland University, Kirrberger Str. Geb. 50, 66421 Homburg, Germany; 2 Department of Urology, Saarland University, Homburg, Germany; 3 Spencer-Fontayne Corporation, Jersey City, NJ USA

**Keywords:** Metastatic castration-resistant prostate cancer (mCRPC), Prostate-specific membrane antigen (PSMA), Lutetium-177-PSMA-617 radioligand therapy (^177^LuPSMA-617 RLT), “Real-world” data, Everyday practice

## Abstract

**Purpose:**

Preliminary data from retrospective analyses and recent data from large randomized controlled trials suggest safety and efficacy of radioligand therapy (RLT) targeting prostate-specific membrane antigen (PSMA) in men with metastatic castration-resistant prostate cancer (mCRPC). Limited data on this modality have been published regarding large samples treated in everyday practice.

**Methods:**

We analyzed prospectively collected registry data regarding lutetium-177 (^177^Lu)-PSMA-617 RLT of 254 consecutive men with mCRPC seen in everyday academic practice. Since ^177^Lu-PSMA-617 was experimental salvage treatment following failure of individually appropriate conventional therapies, patients were generally elderly and heavily pretreated (median age 70 years; prior taxanes 74.0%, 188/254), with late–end-stage disease (visceral metastasis in 32.7%, 83/254). Primary endpoints were response to RLT, defined by changes from baseline serum prostate-specific antigen (PSA) concentration, PSA progression-free survival (PSA-PFS), and overall survival (OS), estimated with Kaplan–Meier statistics, and caregiver-reported and patient-reported safety. Unless noted, median (minimum–maximum) values are given.

**Results:**

Patients received 3 (1–13) ^177^Lu-PSMA-617 activities (6.5 [2.5–11.6] GBq/cycle) every 5.7 (3.0–11.0) weeks. Best response was ≥ 50% PSA reduction in 52.0% of patients (132/254). PSA-PFS was 5.5 (95% confidence interval [95%CI] 4.4–6.6) months and OS, 14.5 (95%CI 11.5–17.5) months. In multivariable Cox proportional-hazards modeling, response to the initial ≤ 2 RLT administrations was the strongest significant prognosticator related to OS (hazard ratio 3.7 [95%CI 2.5–5.5], *p* < 0.001). No RLT-related deaths or treatment discontinuations occurred; the most frequent RLT-related Grade 3/4 adverse events were anemia (18/254 patients, 7.1%), thrombocytopenia (11/254, 4.3%), and lymphopenia (7/254, 2.8%). RLT-related xerostomia, all grade 1/2, was noted in 53/254 (20.9%).

**Conclusions:**

In a large, prospectively observed “real-world” cohort with late-stage/end-stage mCRPC and conventional treatment failure, ^177^Lu-PSMA-617 RLT was effective, safe, and well-tolerated. Early biochemical disease control by such therapy was associated with better OS. Prospective study earlier in the disease course may be warranted.

**Supplementary Information:**

The online version contains supplementary material available at 10.1007/s00259-021-05525-7.

## Introduction

Treatment options to extend disease control and survival in patients with metastatic castration-resistant prostate carcinoma (mCRPC) have expanded dramatically in the past two decades [1–4]. Nonetheless, mCRPC remains lethal and new therapies continue to be much-needed [5].

For this reason, radioligand therapy (RLT) targeting prostate-specific membrane antigen (PSMA) has attracted interest as a potential treatment for mCRPC [6–14]. PSMA is a 750-amino acid transmembrane protein; its function is currently unclear but is hypothesized to involve transport [15]. PSMA is heterogeneously expressed in an array of benign and malignant extraprostatic tissues as well as in prostatic tissue [15–21]. However, expression typically is orders of magnitude greater in cancerous versus healthy prostatic tissue.

A number of small-molecule PSMA ligands have been developed [22]. Among these, the most widely reported for RLT is PSMA-617, introduced in 2013 by the University of Heidelberg Department of Nuclear Medicine and the Clinical Cooperation Unit, Nuclear Medicine, Heidelberg, Germany [23]. Characterized by strong binding affinity to PSMA and highly-efficient internalization into prostate cancer cells, PSMA-617 can be conjugated to the gamma and beta-emitter lutetium-177 (^177^Lu), among other isotopes [23].

Preliminary data suggested safety and efficacy of ^177^Lu-PSMA RLT in men with mCRPC who failed conventional therapies [9, 12, 13]. However, until lately, most published experience, e.g., 13/17 studies included in a recent meta-analysis and systemic literature review [9], involved sample sizes < 50 patients. Very recently, reports [10, 14] have been published regarding two large, prospective, multicenter, randomized controlled trials. The VISION study demonstrated that ^177^Lu-PSMA-617 RLT plus standard care significantly increased OS versus standard care alone [14]; the TheraP study showed that ^177^Lu-PSMA-617 RLT was associated with higher PSA response and fewer grade 3/4 adverse events (AEs) than was cabazitaxel [10]. These studies have provided high-quality evidence of ^177^Lu-PSMA-617 RLT efficacy and safety. However, to our knowledge, limited data have been published regarding these endpoints in large samples of patients treated in everyday practice.

We therefore evaluated, and report here, response, outcomes, and safety associated with ^177^Lu-PSMA-617 RLT in a relatively large cohort of prospectively-observed patients with mCRPC treated in “real-world” routine conditions at an academic center. We also conducted multivariable analyses to identify predictors of biochemical disease control by ^177^Lu-PSMA-617 RLT, as reflected by prostate-specific antigen progression-free survival (PSA-PFS). We further sought to determine whether biochemical disease control by ^177^Lu-PSMA-617 RLT was associated with overall survival (OS).

## Methods

### Patients and ethics

The cohort comprised 254 consecutive men with progressive histologically-confirmed mCRPC who between January 2016 and October 2020, received at least one ^177^Lu-PSMA-617 activity at the University of Saarland Department of Nuclear Medicine. The patients were entered into our Prospective **RE**gistry to **A**ssess Outcome and Toxicity of Targeted Radionuc**LI**de **T**herap**Y** in Patients with mCRPC in Clinical Routine (REALITY Registry). At the close of this analysis (30 March 2021), the patients had complete follow-up data for ≥ 5 months after the first ^177^Lu-PSMA-617 administration. Some data on 47/254 patients (18.5%) were previously reported [12, 24].

Intense tumoral PSMA expression on gallium-68 (^68^ Ga)-PSMA-11 positron emission tomography/computed tomography (PET/CT) (Supplementary Fig. S1) was an eligibility criterion for ^177^Lu-PSMA-617 RLT. Such expression was defined as tracer uptake markedly higher than (physiologic) uptake in healthy liver tissue. Other eligibility criteria were: estimated glomerular filtration rate (eGFR) > 30 mL/min/1.73 m^2^, leukocytes ≥ 2 G/L, platelets > 75 × 10^9^/L, and Eastern Oncology Cooperative Group (ECOG) performance status ≤ 3.

Supplementary Table S1 summarizes study sample characteristics at the start of RLT (“baseline”). Reflecting their late-stage/end-stage disease, the 254 patients tended to be elderly, and had heavy tumor burdens. Nearly all cases had bone involvement, and approximately 33% of cases had visceral metastasis. Additionally, the patients were relatively frequently in poor general condition (ECOG performance status ≥ 2 in almost 40%) and were heavily pretreated, including with one or more taxanes in almost 75% of cases.

The 254 patients represent 79.1% of 321 men screened for ^177^Lu-PSMA-617 RLT; 27 patients (8.4%) were not given RLT due to insufficient ^68^Ga-PSMA-11 uptake on screening PET/CT, and 5 (1.6%), due to preexisting grade 4 thrombocytopenia. Another 35 patients (10.9%) began RLT with ^177^Lu-PSMA-617/actinium-225-PSMA-617 “tandem” combination therapy, and hence were excluded from this analysis.

Establishment and maintenance of the REALITY Registry and analysis of its data starting from January 2016 were approved by our institutional review board, Ärztekammer des Saarlandes/Saarbrücken (decision 140/17). All patient care was given, and ^177^Lu-PSMA-617 RLT and the present study were performed, as mandated in the Declaration of Helsinki and the German Pharmaceutical Act §13 (2b). Patients provided written informed consent for ^177^Lu-PSMA-617 RLT and for use of their de-identified data in scientific publications. The study was registered on clinicaltrials.gov (identifier NCT04833517).

### ^*177*^*Lu-PSMA-617 RLT*

^177^Lu-PSMA-617 was given as experimental salvage therapy, after failure of all conventional treatments appropriate for the given individual. The planned regimen was 4–6 cycles at 6 ± 2-week intervals. However, the number or interval between cycles, or both, could be individualized based on tolerability, tumor burden, and the patient’s general condition. Patients continued on androgen deprivation therapy, zoledronic acid, and denosumab while on RLT.

For each cycle of ^177^Lu-PSMA-617 RLT, patients received a 15–20-min intravenous infusion of the radionuclide while hospitalized in our radioprotection ward. Absent medical contraindications, patients were eligible for discharge after a minimum hospitalization of 48–96 h post-infusion. ^177^Lu-PSMA-617 was labeled using methods analogous to those published elsewhere [25].

### Follow-up

Patients were seen at baseline and within 1 week before subsequent cycles of ^177^Lu-PSMA-617. Patients underwent physical examination and PSA testing at all visits. In this everyday practice setting and in elderly, often frail, late-stage or end-stage patients, conventional CT, ^68^Ga-PSMA-11 PET/CT, and fluouride-18 fluorodexoxyglucose PET/CT were performed only as specifically indicated, rather than routinely. After RLT discontinuation, data regarding PSA concentration, further treatment, and survival status were collected every 3–6 months from the patients, their families, and their urologists or other treating physicians, as applicable.

### Primary endpoints

The primary endpoints of this analysis were ^177^Lu-PSMA-RLT (1) efficacy, as reflected by biochemical response to RLT, PSA-PFS, and OS, and (2) safety, as reflected by incidences of treatment-related AEs by type and severity, and of AE-related treatment discontinuations. Biochemical response to RLT was evaluated based on changes from baseline in the serum PSA level. Biochemical partial response (PR) was defined as a ≥ 50% decrease, and biochemical stable disease (SD), as an intermediate change (< 50% decrease to < 25% increase). Biochemical progressive disease (PD) was defined using Prostate Cancer Clinical Trials Working Group 3 criteria [26] as a relative PSA increase of ≥ 25% and an absolute increase of at least 2 ng/mL.

PSA-PFS comprised the time from the start of ^177^Lu-PSMA-617 RLT until the first of (1) documented biochemical PD; (2) death from any cause; (3) start of another treatment modality, e.g., chemotherapy or “tandem” RLT [24]; or (4) the latest study visit. OS was defined as the interval from the start of ^177^Lu-PSMA-617 RLT to the first of (1) death from any cause, (2) start of another treatment modality, or (3) the latest study visit.

To evaluate RLT safety, blood counts and routine laboratory tests of kidney and liver function were performed at baseline, before each ^177^Lu-PSMA-617 cycle, and approximately monthly after RLT stopped. Laboratory and clinical toxicities were recorded and rated based on caregiver and patient report, using the Common Terminology Criteria for Adverse Events (CTCAE) version 4.03 (https://evs.nci.nih.gov/ftp1/CTCAE/CTCAE_4.03/CTCAE_4.03_2010-06-14_QuickReference_5x7.pdf, last accessed 31 July 2021); the relationship of an AE with RLT was determined subjectively by the investigators. Xerostomia was assessed based on a standardized patient-completed questionnaire developed in-house and administered during hospitalization and at each outpatient visit. Frequency of AE-related treatment discontinuations and treatment-related deaths was tabulated.

### Statistics

PSA-PFS and OS were analyzed using Kaplan–Meier statistics. To identify predictors of each endpoint, Cox proportional-hazards modeling was performed. We used as covariates baseline categorical factors previously considered to influence survival in the mCRPC setting. These factors comprised age, presence/absence of visceral metastasis, ECOG performance status, concentrations of each of PSA, hemoglobin, and alkaline phosphatase (ALP), and histories of each of taxane therapy or 223-radium (^223^Ra; Xofigo, Bayer, Berlin, Germany) therapy [27–31]. To test the association of early biochemical disease control by ^177^Lu-PSMA-617 RLT and OS, early biochemical failure of this modality, i.e., biochemical PD after 1–2 courses, also was considered as a potential determinate of OS. For each factor, univariate regression was performed. Variables contributing to the univariate model (*p* < 0.1) were included in multivariable analysis using a stepwise model by backward elimination. The significance level for all analyses was *p* < 0.05. SPSS version 23 (SPSS Inc., Chicago, IL, USA) and Prism 8 (GraphPad Software, San Diego, USA) were used.

## Results

### ^*177*^*Lu-PSMA-617 RLT*

The median (minimum–maximum) administered ^177^Lu-PSMA-617 activity was 6.5 (2.5–11.6) GBq per cycle and 21.2 (5.1–77.8) GBq cumulatively (Supplementary Table S2). Patients received a median (minimum–maximum) 3 (1–13) cycles of such RLT, given at median (minimum–maximum) intervals of 5.7 (3.0–11.0) weeks. Additional details regarding the RLT regimen are given in Supplementary Table S2.

### Efficacy

Efficacy-related outcomes are summarized in Supplementary Table S3.

### PSA response

**Fig. 1:**
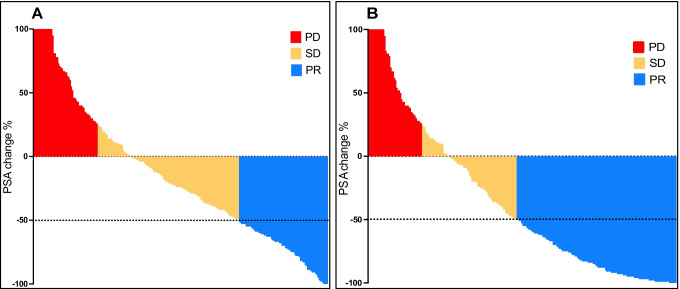
Waterfall plots of **A** PSA responses (percentage change from baseline value) after the first ^177^Lu-PSMA-617 cycle and **B** best PSA responses to the overall course of ^177^Lu-PSMA-617 RLT for the entire cohort (*N* = 254). PD, [biochemical] progressive disease; PR, [biochemical] partial response; RLT, radioligand therapy; SD, [biochemical] stable disease. Please refer to “Methods” section for response category criteria

Figure 1 displays waterfall plots of PSA responses to the first cycle of ^177^Lu-PSMA-617 RLT and of best PSA responses to the entire course of such therapy. At 4–6 weeks after the first cycle, PSA concentration had declined from baseline levels in 169/254 patients (66.5%, 95% confidence interval (CI) 60.5–72.1%), with a ≥ 50% decline in 77/254 (30.3%, 95%CI 25.0–36.2%). Over the entire course of ^177^Lu-PSMA-617 RLT, 132/254 patients (52.0%, 95%CI 45.8–58.0%) had a ≥ 50% decline as their best PSA response.

### PFS-PSA and OS

**Fig. 2:**
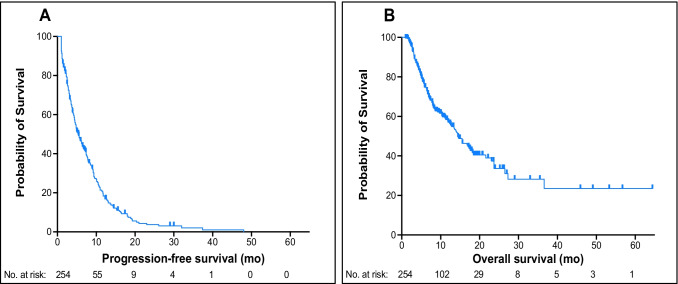
Kaplan–Meier curves of **A** PSA-PFS and **B** OS for the entire cohort (*N* = 254). Median PSA-PFS was 5.5 (95%CI, 4.4–6.6) months, and median OS, 14.5 (95%CI, 11.5–17.5) months. PSA-PFS, prostate-specific antigen progression-free survival; OS, overall survival

Figure 2 shows Kaplan–Meier curves for PSA-PFS and OS for the entire cohort. After a median (minimum–maximum) follow-up of 14.9 (5.0–64.4) months, the median (95% CI) PSA-PFS was 5.5 (4.4–6.6) months, while the median (95% CI) OS was 14.5 (11.5–17.5) months. ^68^Ga-PSMA-11 PET/CT images from patients with prolonged PSA-PFS and OS appear in Supplementary Fig. S1.

**Fig. 3:**
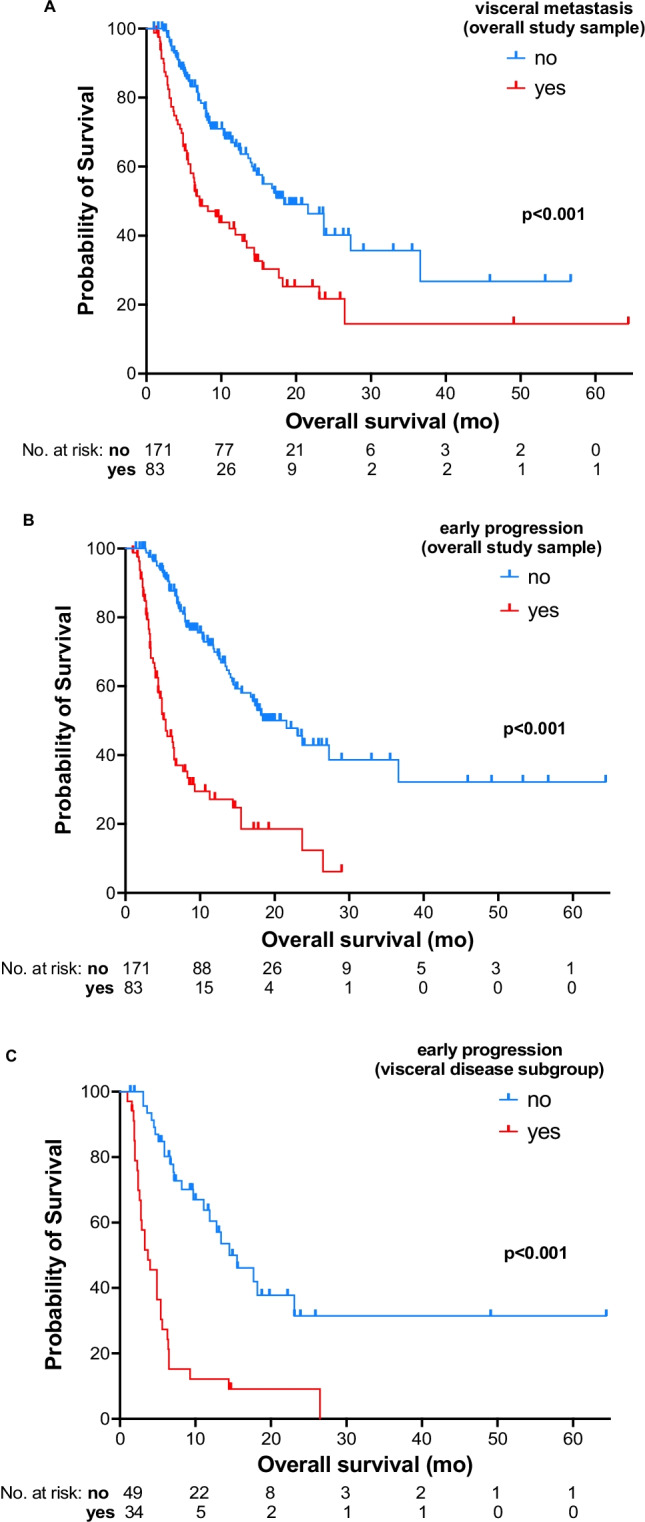
Kaplan–Meier curves for OS stratified by **A** presence or absence of visceral metastasis at the start of ^177^Lu-PSMA-617 therapy (baseline) (overall cohort, *N* = 254); **B** PSA response to the first ≤ 2 courses of ^177^Lu-PSMA-617 (overall cohort, *N* = 254); **C** PSA response to the first ≤ 2 courses of ^177^Lu-PSMA-617 in the subgroup with visceral metastasis at baseline (*n* = 83). As seen in Table 2, presence of visceral metastasis at baseline and PSA progression after the first ≤ 2 courses of ^177^Lu-PSMA-617 were independent negative prognostic factors for OS. Notably, the significant negative prognostic effect of early PSA progression was seen even within the subgroup of patients with visceral metastasis at baseline (median OS, early biochemical progression vs. early biochemical disease control under RLT: 3.7 vs. 14.5 months; univariate HR [95% CI] for shorter survival, early biochemical progression relative to early biochemical disease control: 4.1 [2.1–7.5], *p* < 0.001, log-rank test. OS, overall survival; PSA-PFS, prostate-specific antigen progression-free survival

Univariate and multivariable analyses of potential predictive factors for PSA-PFS or OS are summarized in Tables 1 and 2, respectively. Age ≤ 65 years, ECOG performance status 0–1, baseline ALP < 220 U/L, and absence of taxane pretreatment independently predicted longer PSA-PFS. The second and third of these factors independently predicted longer OS, as did hemoglobin ≥ 10 g/L or absence of visceral metastasis (Fig. 3A) at baseline.

**Table 1:** Relationship of selected baseline factors and PSA-PFS in 254 patients with mCRPC treated with ^177^Lu-PSMA-617 RLT

Variable		PSA-PFS, months Median (95% CI)	Univariate analysis		Multivariable analysis	
	*n* (%)		HR (95% CI)	*p*	HR (95% CI)	*p*
Overall cohort	254 (100.0%)	5.5 (4.4–6.6)				
Age						
≤ 65 years	69 (27.2%)	4.2 (2.9–5.5)	1.4 (1.0–1.8)	0.048	1.4 (1.1–1.9)	**0.016**
> 65 years	185 (72.8%)	6.4 (5.2–7.6)				
Visceral metastases						
Yes	83 (32.7%)	4.1 (2.9–5.4)	1.4 (1.0–1.8)	0.039	-	0.158
No	171 (67.3%)	6.6 (5.4–7.9)				
ECOG performance status						
≥ 2	95 (37.4%)	3.7 (2.9–4.4)	1.3 (1.2–1.5)	< 0.001	1.2 (1.1–1.5)	**0.001**
0–1	159 (62.6%)	7.1 (5.9–8.4)				
PSA, baseline						
≥ 1000 ng/mL	34 (13.4%)	4.9 (4.1–5.7)		0.953		
< 1000 ng/mL	220 (86.6%)	5.5 (4.4–6.6)	-			
Hemoglobin, baseline						
< 10 g/dL	66 (26.0%)	3.7 (2.8–4.6)	1.8 (1.3–2.4)	< 0.001	-	0.216
≥ 10 g/dL	188 (74.0%)	6.2 (4.9–7.4)				
ALP, baseline						
≥ 220 U/L	64 (25.2%)	3.1 (2.1–4.0)	1.9 (1.5–2.7)	< 0.001	1.7 (1.3–2.4)	**0.001**
< 220 U/L	190 (74.8%)	6.7 (5.3–8.0)				
Prior taxanes						
Yes	188 (74.0%)	4.6 (3.9–5.2)	1.4 (1.0–1.9)	0.023	1.4 (1.0–1.9)	**0.031**
No	66 (26.0%)	9.2 (7.0–11.4)				
Prior ^223^Ra therapy						
No	196 (77.2%)	5.1 (4.1–5.9)	-	0.383		
Yes	58 (22.8%)	7.0 (3.9–10.0)				

*p* values from the multivariable analysis that are in bold type are statistically significant at *p* < 0.05

^223^Ra radium-223, *ALP* alkaline phosphatase, *CI* confidence interval, *ECOG* Eastern Cooperative Oncology Group, *HR* hazard ratio, *mCRPC* metastatic castration-resistant prostate cancer, *PSA* prostate-specific antigen, *PSA-PFS* prostate-specific antigen progression-free survival, *RLT* radioligand therapy

**Table 2:** Relationship of OS with early biochemical response to RLT and with baseline factors in patients with mCRPC treated with ^177^Lu-PSMA-617

Variable		OS, months Median (95% CI)	Univariate analysis		Multivariable analysis	
	*n* (%)		HR (95% CI) for mortality	*p*	HR (95% CI) for mortality	*P*
Overall cohort	254	14.5 (11.5–17.5)				
Early biochemical failure (PD after ≤ 2 cycles of RLT)						
Yes	83 (32.7%)	5.4 (3.8–6.9)	3.8 (2.6–5.5)	< 0.001	3.7 (2.5–5.5)	** < 0.001**
No	171 (67.3%)	21.6 (16.2–26.9)				
Age						
≤ 65 years	69 (27.2%)	11.8 (5.8–17.7)	-	0.098		
> 65 years	185 (72.8%)	15.5 (11.5–19.5)				
Visceral metastasis						
Yes	83 (32.7%)	7.1 (3.9–10.3)	2.2 (1.5–3.2)	< 0.001	1.5 (1.1–2.3)	**0.022**
No	171 (67.3%)	18.4 (12.8–24.0)				
ECOG performance status						
≥ 2	95 (37.4%)	6.9 (4.9–8.9)	1.7 (1.4–2.1)	< 0.001	1.4 (1.1–1.7)	**0.004**
0–1	159 (62.6%)	23.7 (14.9–32.4)				
Baseline PSA						
≥ 1000 ng/mL	34 (13.4%)	12.4 (4.1–20.7)		0.497		
< 1000 ng/mL	220 (86.6%)	14.7 (11.9–17.4)	-			
Hemoglobin, baseline						
< 10 g/dL	66 (26.0%)	5.6 (4.0–7.2)	3.5 (2.4–5.2)	< 0.001	1.9 (1.2–3.0)	**0.008**
≥ 10 g/dL	188 (74.0%)	18.4 (13.4–23.4)				
ALP, baseline						
≥ 220 U/L	64 (25.2%)	5.9 (4.1–7.7)	3.1 (2.1–4.6)	< 0.001	2.0 (1.3–3.1)	**0.001**
< 220 U/L	190 (74.8%)	18.4 (12.6–24.2)				
Prior taxanes						
Yes	188 (74.0%)	13.4 (10.4–16.5)	1.6 (1.0–2.6)	0.029	-	0.389
No	66 (26.0%)	21.6 (13.6–29.5)				
Prior ^223^Ra therapy						
No	196 (77.2%)	14.3 (9.9–18.7)	-	0.369		
Yes	58 (22.8%)	15.5 (11.7–19.3)				

*p* values from the multivariable analysis that are in bold type are statistically significant at *p* < 0.05

^177^Lu lutetium-177, ^*223*^*Ra* radium-223, *ALP* alkaline phosphatase, *CI* confidence interval, *ECOG* Eastern Cooperative Oncology Group, *HR* hazard ratio, *mCRPC* metastatic castration-resistant prostate cancer, *OS* overall survival, *PSA* prostate-specific antigen, *PSMA* prostate-specific membrane antigen, *RLT* radioligand therapy

Notably, biochemical PD after 1–2 courses of ^177^Lu-PSMA-617 RLT was an independent predictor of shorter OS in the overall study sample (Table 2; Fig. 3B). Indeed, based on HR, early response to ^177^Lu-PSMA-617 RLT was the strongest studied prognostic factor related to OS. Of interest, the apparent positive prognostic effect of early biochemical disease control by RLT held true even within the subgroup with visceral metastasis at baseline (Fig. 3C).

### AEs and safety

^177^Lu-PSMA-617 appeared to be safe and generally well-tolerated in this heavily-pretreated, elderly patient population with late-stage/end-stage disease. No deaths were attributed to RLT and no patient discontinued RLT due to AEs. Aditionally, the four most common AEs judged to be at least possibly related to ^177^Lu-PSMA-617 RLT, namely, anemia, fatigue, thrombocytopenia, and lymphopenia, were observed in Grade 3/4 severity in only 7.1% (18/254), 2.4% (6/254), 4.3% (11/254), and 2.8% (7/254) of patients, respectively (Table 3). Of interest, xerostomia, the fifth most common ^177^Lu-PSMA-617 RLT side effect, was absent in almost 80% of patients, was noted only in Grade 1/2 severity, and was generally transient.

**Table 3:** Patient incidence and severity of treatment-related^a^ AEs observed over the course of ^177^Lu-PSMA-617 RLT in 254 patients with mCRPC

AE	Total		Grade 1/2^b^		Grade 3/4^b^	
	*N*	% (95%CI)	*n*	% (95%CI)	*n*	% (95%CI)
Any	212	83.5 (78.4–86.5)	191	75.2 (69.5–80.1)	21	8.3 (5.5–12.3)
Anemia	81	31.9 (26.5–37.9)	63	24.8 (19.9–30.5)	18	7.1 (4.5–10.9)
Fatigue	78	30.7 (25.4–36.6)	72	28.3 (23.2–34.2)	6	2.4 (1.1–5.1)
Thrombocytopenia	57	22.4 (17.7–28.0)	46	18.1 (13.9–23.3)	11	4.3 (2.4–7.6)
Leukopenia	57	22.4 (17.7–28.0)	50	19.7 (15.3–25.0)	7	2.8 (1.3–5.6)
Xerostomia	53	20.9 (16.3–26.3)	53	20.9 (16.3–26.3)	0	0
Anorexia	32	12.6 (9.1–17.2)	32	12.6 (9.1–17.2)	0	0
eGFR abnormalities	29	11.4 (8.1–15.9)	29	11.4 (8.1–15.9)	0	0
Weight loss	24	9.4 (6.4–13.7)	24	9.4 (6.4–13.7)	0	0
Nausea	8	3.1(1.6–6.1)	8	3.1 (1.6–6.1)	0	0
Vomiting	5	1.9 (0.8–4.5)	5	1.9 (0.8–4.5)	0	0
Others	9	3.5 (1.9–6.6)	9	9 (1.9–6.6)	0	0

^177^Lu lutetium-177, *AE* adverse event, *CI* confidence interval, *CTCAE* Common Terminology Criteria for Adverse Events, *eGFR* estimated glomerular filtration rate, *mCRPC* metastatic castration-resistant prostate cancer, *PSMA* prostate-specific membrane antigen, *RLT* radioligand therapy

^a^Relationship of an AE to ^177^Lu-PSMA-617 was assessed subjectively by the investigators

^b^Severity of toxicities was classified using the CTCAE version 4.03 (https://evs.nci.nih.gov/ftp1/CTCAE/CTCAE_4.03/CTCAE_4.03_2010-06-14_QuickReference_5x7.pdf, last accessed 31 July 2021)

## Discussion

This registry study is, to our knowledge, the largest yet published of PSMA-targeted RLT in everyday practice and has the additional strength of relying on prospective observation. Our findings are notable in providing evidence of the encouraging efficacy and safety of ^177^Lu-PSMA-617 RLT even in “real-world” patients. Unsurprisingly, our patients appear to have had less favorable baseline characteristics, and to have received a less intense RLT regimen, compared to the active treatment groups in the multicenter, randomized controlled VISION and TheraP trials [10, 14].

Compared to VISION’s active treatment group (n = 551), our patients had greater prevalence of visceral metastasis (33% vs < 20%) and of markedly impaired general condition (ECOG performance status ≥ 2 in 37% vs 7% of patients). Our sample also had higher median baseline PSA and ALP levels (Table 4), while the cohorts’ histories of prior therapy appeared to be roughly alike [14]. VISION’s active treatment group received a median (minimum–maximum) cumulative ^177^Lu-PSMA-617 activity of 37.5 (7.0–48.3) GBq in 5 (1–6) cycles, versus 21.2 (5.1–77.8) GBq in 3 (1–13) administrations for our patients. Despite these differences, our study found a rate of ≥ 50% PSA decrease (52% vs. 46%) and median OS (14.5 vs. 15.3 months) that greatly resembled those of VISION patients given RLT.

**Table 4:** Comparison of key characteristics and results from the most important studies of ^177^Lu-PSMA-617 RLT in mCRPC highlighting efficacy in the post-taxane setting

	REALITY	VISION [14]	TheraP [10]
Number of patients (^177^LuPSMA617 arm)	254	551	99
Median follow-up	14.9 months	20.3 months	18.4 months
Molecular imaging inclusioncriteria	None	None	No FDG + /PSMA − sites
Patient characteristics(post-taxane cohorts)			
Previous taxane (≥ 1 regimen)	n=188	n=551	n=99
Previous 2nd line cabazitaxel	32.7% (83)	37.9% (209)	0%
Impaired condition(ECOG ≥ 2)	40.4%	7.4%	4%
Visceral metastasis	28.4%	< 20%^a^	7%
Liver metastasis	21.9%	11.4%	n.a
Median ALP, U/L	135.0	105.0	111.0
Median PSA, ng/mL	182	76	93
RLT regimen			
Median single activities Median cumulative activity	6.5 GBq q 6 weeks21.2 (5.1–77.8) GBq	6.9 GBq q 6 weeks37.5 (7.0–48.3) GBq	6–8.5 GBq q 6 weeks^b^n.a
Results(post-taxane cohorts)			
Best PSA response, > 50% decrease	48%	46%^c^	66%
PSA-PFS (median)	4.5 months	n.a. (8.7 mo imaging-based PFS)	5.1 months
OS (median)	13.4 months	15.3 months	Not yet reported
Toxicity/AEs			
Xerostomia, grades1–2	20.9%	38.8%	60%
Thrombocytopenia,grades 3–4	4.3%	7.9%	11%
Leukopenia, grades3–4	2.8%	2.5%	1%

*ALP* alkaline phosphatase, *ECOG* Eastern Cooperative Oncology Group, *FDG* fluouride-18 fluorodexoxyglucose, *PSA* prostate-specific antigen, *PSMA* prostate-specific membrane antigen, *RLT* radioligand therapy, *n.a.* not available

^a^Clear data were not reported regarding overall prevalence of visceral metastases: liver metastases were present in 11.4%, lung metastases in 8.9%, but it was not reported how many patients had both

^b^This was the planned activity, actual administered activity not available

^c^Only reported for 385 patients

Compared to TheraP’s active treatment group (*n* = 99), our patients had a much greater prevalence of visceral metastasis as well ECOG ≥ 2 status, and had higher median baseline PSA and ALP. However, since TheraP used cabazitaxel as the comparator for RLT, our patients were more heavily pre-treated. The planned regimen in TheraP’s active treatment arm (43.5 GBq total in 6 administrations), envisioned a substantially higher cumulative activity in more cycles than were given to our patients. Nonetheless, the biochemical PR rate in the TheraP active treatment patients did not dramatically differ from ours (66% vs 52%).

Lower rates of grade 3/4 AEs were recorded in our patients than in VISION’s active treatment arm [14]. Additionally, while no toxicity-related ^177^Lu-PSMA-617 discontinuations or treatment-related deaths were seen in our cohort, there were 11.9% and 3.6% rates of these phenomena, respectively, in VISION’s RLT group. One may speculate that there were two important reasons for these differences. First, the rates reported by the VISION investigators appear to be for treatment-*emergent* rather than treatment-*related* toxicity, which is what we report. Secondly, as noted above, the VISION patients typically received more intense and lengthier ^177^Lu-PSMA-617 treatment than did our patients.

Interestingly, the ^177^Lu-PSMA-617 safety and tolerability profile in the TheraP active treatment group, appeared more like that in our patients (bearing in mind that again, treatment-*emergent* toxicity is being compared with treatment-*related* AEs). Most notably, TheraP had only a 1% rate of toxicity-related ^177^Lu-PSMA-617 discontinuation and no treatment-related deaths. Regarding the comparison of RLT safety in the two studies, one may speculate that the more intense RLT regimen in TheraP may have been counterbalanced by the less heavily pretreated nature of their active treatment group.

A notable efficacy-related finding of our study was that even after statistical adjustment for patient, disease, and prior treatment characteristics, early biochemical disease control by ^177^Lu-PSMA-617 RLT was significantly associated with OS; this finding confirmed and extended a similar, earlier, novel observation in a 28-patient subgroup of the present cohort, all of whom had liver metastasis [12]. Indeed, early biochemical progression was the strongest studied determinate related to OS in the 254-patient multivariable analysis, with a HR for mortality nearly double or more than double that of the other 4 significant negative prognostic factors identified (Table 2, Fig. 3B). The relationship of biochemical response to PSMA-targeted RLT with OS may suggest cause and effect regarding ^177^Lu-PSMA-617 treatment and the relatively lengthy OS in our late-stage/end-stage patients. This hypothesis is supported by the findings regarding comparative efficacy of ^177^Lu-PSMA-617 versus standard care in VISION [14] or versus cabazitaxel in TheraP [10].

Aligned with observations by others [7], another finding of our study that merits mention is that although patients with taxane failure (*n* = 188) benefitted from ^177^Lu-PSMA-617, RLT appeared to be more effective in patients who were taxane-naïve (*n* = 66) (Supplementary Table S5). For example, median (95%CI) PSA-PFS for the respective subgroups was 4.6 (3.9–5.2) months versus 9.2 (7.0–11.4) months, a difference that was statistically significant; median OS was 13.4 (10.4–16.5) months versus 21.6 (13.6–29.5) months, albeit this difference did not attain statistical significance. The seemingly better survival outcomes in the taxane-naïve subgroup may at least partly stem from their less aggressive disease course (reflected by significantly older age and lower prevalence of visceral metastasis) and more intact red marrow compartment (reflected by significantly higher median baseline hemoglobin concentration). Indeed, in our multivariable analysis, history of taxane therapy was not associated with OS (Table 2), although such history was significantly linked with PSA-PFS (Table 1). Nonetheless, our observations regarding taxane pretreatment, coupled with the seemingly comparable activity but much lesser toxicity of ^177^Lu-PSMA-617 RLT relative to other recently-introduced systemic agents, suggest that PSMA-targeted RLT merits formal study earlier in the mCRPC disease course. This hypothesis also is supported by the multivariable association in our study of early biochemical disease control and longer OS, and, more concretely, by TheraP’s observations of greater biochemical effect and lesser toxicity of ^177^Lu-PSMA-617 versus cabazitaxel [10].

We found that older age, better general condition (lower ECOG performance status), and lower baseline serum ALP concentration, also were significant independent determinates of longer PSA-PFS (Table 1). One may speculate that older age and better ECOG performance status may to at least some extent be surrogates for earlier-stage or less-aggressive disease, or less pretreatment. Serum ALP concentration is a marker of bone involvement, and thus when elevated, may reflect advanced-stage or more aggressive disease. One may hypothesize that the negative prognostic significance of taxane pretreatment regarding biochemical response to ^177^Lu-PSMA-617 RLT relates to the prior therapy selecting more resistant or aggressive clones.

Notable limitations of our study should be acknowledged. The most important include lack of a control arm, as well as the single-center nature of the REALITY Registry. Additionally, no fixed-activity protocol was used for ^177^Lu-PSMA-617 RLT. Instead,^177^Lu-PSMA-617 activities, and the number of and intervals between RLT cycles were individually chosen based on total tumor burden, sites of metastases, and the patient’s condition including ECOG performance status and organ function. While this strategy increased individualization of therapy, the variability in RLT regimens arguably renders our overall results harder to interpret. Another limitation of our work is that besides OS, this report does not consider efficacy endpoints unrelated to PSA, such as structural or molecular imaging, pain control, or quality-of-life changes. Regarding structural or molecular imaging, accurate response assessment according to Response Criteria in Solid Tumors [32] was not possible, because CT was performed without contrast enhancement and post-therapy restaging was done at different intervals. Also, in everyday care of typically elderly, frail, late-stage or end-stage patients undergoing an experimental treatment, use of PSA was felt to comprise the least invasive, logistically-easiest, and most rapidly-collected efficacy biomarker. The utility of PSA as such is supported by the significant, strong prognostic power regarding OS of early biochemical disease control in our multivariable analysis.

In conclusion, this, the largest-yet-published report of a prospectively-observed cohort with late–end-stage mCRPC, conventional treatment failure, and care in an everyday setting, provides encouraging evidence that ^177^Lu-PSMA-617 RLT is effective, safe, and well-tolerated outside of clinical trial conditions, and in patients with frequently more advanced disease and heavier pretreatment than those in VISION or TheraP [10, 14]. Early biochemical disease control (PR/SD) after the initial ≤ 2 cycles of RLT was strongly and significantly associated with better OS, even in the presence of negative prognostic factors, e.g., liver metastases. Prospective study of RLT earlier in the disease course may be warranted.

## Supplementary Information

Below is the link to the electronic supplementary material.

Supplementary file1 (DOCX 5.58 MB)

## Abbreviations

^68^Ga: Gallium-68
^153^Sm: Samarium-153
^177^Lu: Lutetium-177
^223^Ra: Radium-223
ADT: Androgen deprivation therapy
AE: Adverse event
ALP: Alkaline phosphatase
CI: Confidence interval
CT: Computed tomography
CTCAE: Common Terminology Criteria for Adverse Events
ECOG: Eastern Cooperative Oncology Group
eGFR: Estimated glomerular filtration rate
HR: Hazard ratio
mCRPC: Metastatic castration-resistant prostate cancer
OS: Overall survival
PD: [Biochemical] progressive disease
PET: Positron emission tomography
PR: [Biochemical] partial response
PSA: Prostate-specific antigen
PSA-PFS: Prostate-specific antigen progression-free survival
PSMA: Prostate-specific membrane antigen
RLT: Radioligand therapy
SD: [Biochemical] stable disease
