# Supplementary material for: 177 Lu-PSMA-617 radioligand therapy of metastatic castration-resistant prostate cancer: Initial 254-patient results from a prospective registry (REALITY Study)
Source: Eur J Nucl Med Mol Imaging. 2021 Sep 7;49(3):1075–85. doi: 10.1007/s00259-021-05525-7 (PMC8803625; doi:10.1007/s00259-021-05525-7)
Supplement: Supplementary file 1 — Supplementary file1 (DOCX 5.58 MB) [file 259_2021_5525_MOESM1_ESM.docx]

**SUPPLEMENTARY TABLES**

# **Supplementary Table S1.** Patient and treatment characteristics

| **Characteristic** | | ***n, minimum–maximum, or %*** |  |
| --- | --- | --- | --- |
| **Total** | | 254 |  |
| **Age** | |  |  |
| Median (minimum—  maximum) — yr. | | 70 (47–88) |  |
| >65 yr — % (n) | | 72.8% (185) |  |
| **Site of metastasis, % (n)** | |  |  |
| Bone  Lymph node | 94.9% (241)  65.4% (166) | |  |
| Any visceral site | 32.7% (83) | |  |
| Liver | | 19.3% (49) |  |
| Lung | | 15.0% (38) |  |
| Brain | | 2.8% (7) |  |
| Other | | 7.5% (19) |  |
| **ECOG performance status, % (n)** | |  |  |
| 0 | | 23.2% (59) |  |
| 1 | | 39.4% (100) |  |
| ≥2 | | 37.4% (95) |  |
| **PSA, baseline** | |  |  |
| Median (minimum–maximum)  - ng/mL | | 167 (0.5—9579) |  |
| >1000 ng/mL | | 13.4% (34) |  |
| **Hemoglobin, baseline** | |  |  |
| Median (minimum–maximum)   - g/dL | | 11.1 (5.0–16.0) |  |
| <10 g/dL | | 26.0% (66) |  |
| **ALP, baseline** | |  |  |
| Median (minimum–maximum)  – U/L | | 128 (27–1806) |  |
| ≥220 U/L | | 25.2% (64) |  |
| **Prior treatments, % (n)** | |  |  |
| ADT | | 100% (254) |  |
| Abiraterone | | 66.1% (168) |  |
| Enzalutamide | | 70.9% (180) |  |
| Abiraterone or  Enzalutamide | | 90.2% (229) |  |
| Abiraterone +  Enzalutamide | | 45.3% (115) |  |
| Docetaxel | | 74.0% (188) |  |
| Second-line cabazitaxel | | 32.7% (83) |  |
| ^223^Ra | | 22.8% (58) |  |
| ^153^Sm | | 4.3% (11) |  |

^153^Sm: samarium-153; ^223^Ra: radium-223; ADT: androgen deprivation therapy; ALP: alkaline phosphatase; ECOG: Eastern Cooperative Oncology Group; PSA: prostate-specific antigen

**Supplementary Table S2.** ^177^Lu-PSMA-617 RLT characteristics (N=254)

| ***Variable*** | ***Median*** | ***Minimum*** | ***Maximum*** | ***Mean ± SD*** |
| --- | --- | --- | --- | --- |
| Number of ^177^Lu-PSMA-617 activities | 3 | 1 | 13 | 4 ± 2 |
| Weeks between ^177^Lu-PSMA-617 cycles | 5.7 | 3.0 | 11.0 | 5.7 ± 1.6 |
| ^177^Lu-PSMA-617 administered activity per  cycle, GBq | 6.5 | 2.6 | 11.6 | 6.5 ± 1.5 |
| Cumulative ^177^Lu-PSMA-617 administered activity, GBq | 21.2 | 5.1 | 77.8 | 24 ± 14 |

^177^Lu: lutetium-177; PSMA: prostate-specific membrane antigen; RLT: radioligand therapy; SD: standard deviation.

**Supplementary Table S3.** Patient outcomes (N=254)

| ***Variable*** | ***Value*** |
| --- | --- |
| Duration of follow-up, months  Median (minimum–maximum) | 14.9 (5.0–64.4) |
| Best PSA response to entire course of  RLT: percentage change from baseline,  % (n)  ≥50% decrease (PR)  <50% decrease to <25% increase (SD)  ≥25% increase (PD) | 52.0% (132)  31.5% (80)  17.3% (44) |
| PSA-PFS, months  median (95% CI) | 5.5 (4.4–6.6) |
| OS, months  median (95% CI) | 14.5 (11.5–17.5) |

^177^Lu: lutetium-177; CI: confidence interval; mCRPC: metastatic castration-resistant prostate cancer; OS: overall survival; PD: [biochemical] progressive disease; PR: partial [biochemical] response; PSA: prostate-specific antigen; PSA-PFS: prostate-specific antigen progression-free survival; PSMA: prostate-specific membrane antigen; RLT: radioligand therapy; SD: [biochemical] stable disease.

**Supplementary Table S4.** Baseline patient characteristics by history of taxane therapy

| ***Characteristic*** | ***Prior taxanes (n=188)*** | ***Taxanes-naïve***  ***(n=66)*** | ***p, Mann-Whitney u test*** |
| --- | --- | --- | --- |
| Age, yr,  Median (25^th^–75^th^  percentiles) | 69 (64–76) | 75 (67–80) | **0.001** |
| Patients with visceral  metastasis, % (n) | 36.7% (69/188) | 21.2% (14/66) | **0.022** |
| Patients with bone  metastasis, % (n) | 95.7% (180/188) | 92.4% (61/66) | 0.332 |
| ECOG performance status, %  (n)  0  1  ≥2 | 20.7% (39/188)  39.4% (74/188)  39.9% (75/188) | 30.3% (20/66)  39.4% (26/66)  30.3% (20/66) | 0.167 |
| PSA, ng/mL, median  (25^th^–75^th^ percentiles) | 177 (51.3–551.3) | 144.5 (59–737) | 0.800 |
| Hemoglobin, g/dL, median  (25^th^–75^th^ percentiles) | 11.0 (9.5–11.0) | 12.0 (10.0–13.0) | **0.021** |
| ALP, U/L, median (25^th^–75^th^  percentiles) | 130.5 (76.0–224.3) | 127.5 (79.0–227.7) | 0.995 |
| Platelets, 10^9^/L, median  (25^th^–75^th^ percentiles) | 230 (175–298) | 218 (170–265) | 0.349 |
| eGFR, mL/min/1.73 m^2^ | 84.0 (67.5–93.0) | 79.0 (63.7–89.3) | 0.099 |
| Prior enzalutamide, % (n) of  patients | 73.4% (138/188) | 63.6% (42/66) | 0.157 |
| Prior abiraterone, % (n) of  patients | 67.6% (127/188) | 62.1% (41/66) | 0.451 |
| Prior  ^223^Ra, % (n) of patients | 22.9% (43/188) | 22.7% (15/66) | 1.000 |

*p* values that are in bold type are statistically significant at *p*<0.05.

^223^Ra: radium-223; ALP: alkaline phosphatase; ECOG: Eastern Cooperative Oncology Group; eGFR: estimated glomerular filtration rate; PSA: prostate-specific antigen; RLT: radioligand therapy

**SUPPLEMENTARY FIGURES**

# **Supplementary Figure S1**

#

Supplementary Figure S1. ^68^Ga-PSMA-11 PET/CT maximum intensity projection images of three patients with sharp decreases in PSA and prolonged PFA-PFS and OS under ^177^Lu-PSMA-617 RLT. Images (A) at baseline and (B) 6 weeks after 6 cycles of ^177^Lu- PSMA-617 RLT in a 67-year-old man with mCRPC who had failed abiraterone, enzalutamide, docetaxel, cabazitaxel, and ^223^Ra. A 99.6% PSA decline from baseline was reflected by an extensive remission of initially diffuse bone metastases on molecular imaging. The patient had remained in biochemical remission for 23 months and was still alive as of the close of the present analysis (follow-up: 27 months). Images (C) at baseline and (D) 6 weeks after 4 cycles of RLT in a 68-year-old patient with mCRPC who had failed docetaxel, abiraterone, an initial course of and a re-challenge with cabazitaxel, and enzalutamide. In this case, a 99.7% PSA decline from baseline was reflected by remission of the bulk of lymph node metastases on molecular imaging. The patient had remained in biochemical remission for 15.5 months, and was still alive as of the close of the present analysis (follow-up: 15.5 months). Images (E) at baseline and (F) 6 weeks after 3 cycles of RLT in a 81-year-old patient with mCRPC who had failed abiraterone, enzalutamide, and docetaxel, and in whom cabazitaxel was contra-indicated due to clinical deterioration. In this case, a 99.9% PSA decline from baseline was reflected by remission of liver and bone metastases on molecular imaging. The patient had remained in biochemical remission for 19.3 months and was still alive as of the close of the present analysis (follow-up: 64.4 months).
